# Calpain 2-mediated autophagy defect increases susceptibility of fatty livers to ischemia–reperfusion injury

**DOI:** 10.1038/cddis.2016.66

**Published:** 2016-04-14

**Authors:** Q Zhao, Z Guo, W Deng, S Fu, C Zhang, M Chen, W Ju, D Wang, X He

**Affiliations:** 1 Organ Transplant Center, The First Affiliated Hospital, Sun Yat-sen University, Guangzhou, China; 2 Biotherapy Department, Sun Yat-sen Memorial Hospital, Sun Yat-sen University, Guangzhou, China

## Abstract

Hepatic steatosis is associated with significant morbidity and mortality after liver resection and transplantation. This study focuses on the role of autophagy in regulating sensitivity of fatty livers to ischemia and reperfusion (I/R) injury. Quantitative immunohistochemistry conducted on human liver allograft biopsies showed that, the reduction of autophagy markers LC3 and Beclin-1 at 1 h after reperfusion, was correlated with hepatic steatosis and poor survival of liver transplant recipients. In animal studies, western blotting and confocal imaging analysis associated the increase in sensitivity to I/R injury with low autophagy activity in fatty livers. Screening of autophagy-related proteins showed that Atg3 and Atg7 expression levels were marked decreased, whereas calpain 2 expression was upregulated during I/R in fatty livers. Calpain 2 inhibition or knockdown enhanced autophagy and suppressed cell death. Further point mutation experiments revealed that calpain 2 cleaved Atg3 and Atg7 at Atg3Δ92–97 and Atg7Δ344–349, respectively. *In vivo* and *in vitro* overexpression of Atg3 or Atg7 enhanced autophagy and suppressed cell death after I/R in fatty livers. Collectively, calpain 2-mediated degradation of Atg3 and Atg7 in fatty livers increases their sensitivity to I/R injury. Increasing autophagy may ameliorate fatty liver damage and represent a valuable method to expand the liver donor pool.

Organ shortage is a critical problem restricting the practice of liver transplantation. Thousands of patients die while on the waiting list, which has prompted the use of ‘marginal donor' livers.^1^ Steatotic livers represent a major component of the marginal donor livers. In western countries, studies have found that 30% of donor livers are steatotic, which has been associated with relatively poor transplant outcomes. The 2-year posttransplant primary graft failure rate and recipient survival rate were 13% and 77%, respectively, in patients receiving fatty livers, compared with a corresponding 3% and 91% in patients using normal livers.^2, 3^ Increased vulnerability of steatotic livers to ischemia–reperfusion (I/R) injury is the major cause of inferior outcomes in transplants using fatty livers. However, the underlying mechanisms are not yet fully understood.^4^

Autophagy is an intracellular lysosomal degradative process operating in the homeostatic clearance of organelles and protein aggregates and is considered an adaptive response to stress or I/R injury. During I/R, autophagy is upregulated by inflammatory mediators, such as tumor necrosis factor-*α* (TNF-*α*) and reactive oxygen species (ROS), and protects hepatocytes from injury by clearing dysfunctional mitochondria, degrading misfolded proteins and generating adenosine triphosphate (ATP). These actions consequently limit ROS generation, relieve endoplasmic reticulum (ER) stress and restore cellular energy.^5^ Impaired autophagy can result in the accumulation of damaged mitochondria, leading to the release of cell death-signaling molecules from one mitochondrion to neighboring mitochondria, rapidly propagating this injurious signaling cascade throughout the cell.^6^ The failure of autophagy to remove even a small subset of damaged mitochondria during I/R can therefore have a significant impact on hepatocellular viability. Therefore, autophagy is essential for hepatic function and cell survival during I/R.

The involvement of autophagy in the pathogenesis of nonalcoholic fatty liver disease was first suggested by the finding that autophagy mediates the breakdown of intracellular lipids in hepatocytes.^7^ Hepatocyte-specific knockout of autophagy-related protein (Atg) 7 fed with a high-fat diet developed markedly increased liver triglyceride and cholesterol content, indicating that defects in autophagy can promote hepatic steatosis. High lipid levels can also affect autophagy activity.^7^ Hyperinsulinemia may contribute to downregulation of autophagy through the Akt/protein kinase B (PKB) pathway in fatty liver.^8^ In addition, autophagy is suppressed in steatotic livers by mammalian target of rapamycin (mTOR) overactivation as a result of an increased amino-acid concentration following overnutrition.^9^ Therefore, a harmful cycle may exist in which independent factors promote both reduced autophagy and hepatic steatosis, and that the decrease in autophagy exacerbates steatosis, further impairing autophagy.

The progression to inflammation and hepatocellular injury marks the development of fatty liver disease. Autophagy may reduce cell injury by regulating cell death pathways triggered by oxidants and TNF, which mediate steatotic liver injury.^10^ However, the role of autophagy in fatty livers during I/R injury is still unknown. In this study, we investigated whether steatotic hepatocytes more sensitive to I/R injury is due to defects in autophagy. Our results indicate that calpain 2-mediated loss of Atg3 and Atg7 contributes to the susceptibility of fatty livers to I/R injury.

## Results

### Decreased LC3 and Beclin-1 expression correlates with human liver allograft steatosis and transplant outcomes

**Figure 1:**
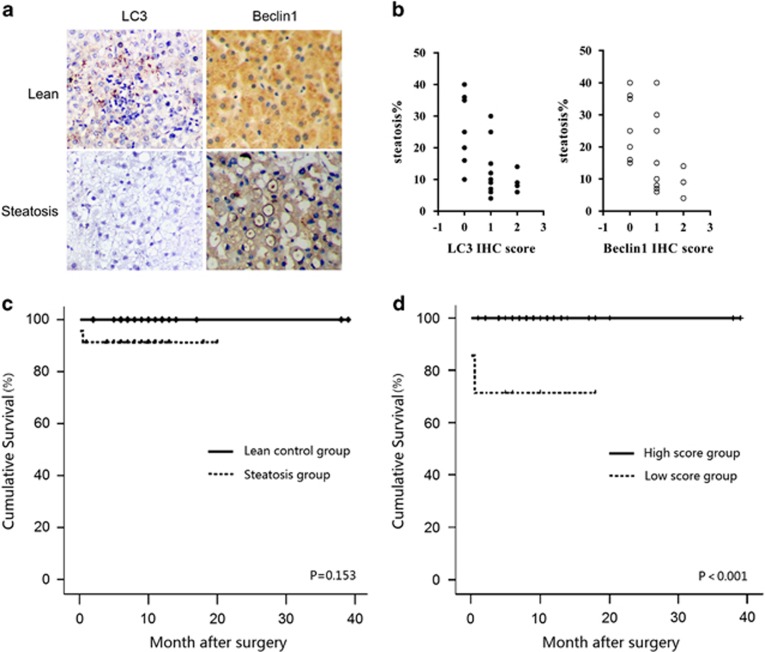
Low expression of LC3 and Beclin-1 in reperfused human steatotic liver allografts is associated with degree of steatosis and poor transplant outcome. (**a**) Human steatotic and control liver samples taken after 1 h of reperfusion during liver transplant surgery were analyzed by IHC. Autophagy markers LC3 and Beclin-1 displayed low expression in steatotic liver. (**b**) Pearson correlation test was used for analysis of correlations between LC3 (left) and Beclin-1 (right) expression score and percentage of steatosis. (**c**) Kaplan–Meier analysis of the data from 46 cases of liver transplantation recipients in survival rates between the steatotic liver allograft group (*n*=23) and the lean control liver allograft group (*n*=23). (**d**) Kaplan–Meier survival curves of overall survival in all transplant patients according to the summed LC3 and Beclin-1 expression score. Low expression score: sum of LC3 and Beclin-1 score ≤1. High expression score: sum of LC3 and Beclin-1 score >1

To investigate the correlation of autophagy with human liver I/R injury, we detected the expression of autophagy markers LC3 and Beclin-1 in 23 steatotic liver allograft samples and 23 normal controls by immunohistochemical (IHC) staining (patients' pretransplant clinical data are summarized in Supplementary Table 1). Liver samples were obtained at 1 h after reperfusion during transplantation. Compared with normal controls, the fatty livers expressed significantly lower levels of LC3 and Beclin-1 (Figure 1a). The expression scores of LC3 (*r*=−0.62, *P*<0.05) and Beclin-1 (*r*=−0.54, *P*<0.05) were inversely correlated with the percentages of steatosis (Figure 1b).

Importantly, we documented a higher incidence of posttransplant early allograft dysfunction, as described by Olthoff *et al*,^11^ in the fatty liver group when compared with the control group (73.9% *versus* 30.4%, *P*<0.05). However, the overall cumulative survival rates between the two groups were not significantly different (*P*=0.153; Figure 1c). We further evaluated whether the summed expression scores of LC3 and Beclin-1 in liver allografts correlated with transplant outcomes in all 46 patients. We observed that low expression score (≤1) predicted shorter cumulative overall survival (*P*<0.001; Figure 1d). Furthermore, in the fatty liver group, low expression score (≤ 1) was also correlated with poor transplant outcomes (*P*<0.05; Supplementary Figure 1). These results indicate that reduced expression of LC3 and Beclin-1 are associated with severity of liver allograft steatosis and unfavorable transplant outcomes.

### Fatty livers are more susceptible to I/R injury *in vivo* and *in vitro*

**Figure 2:**
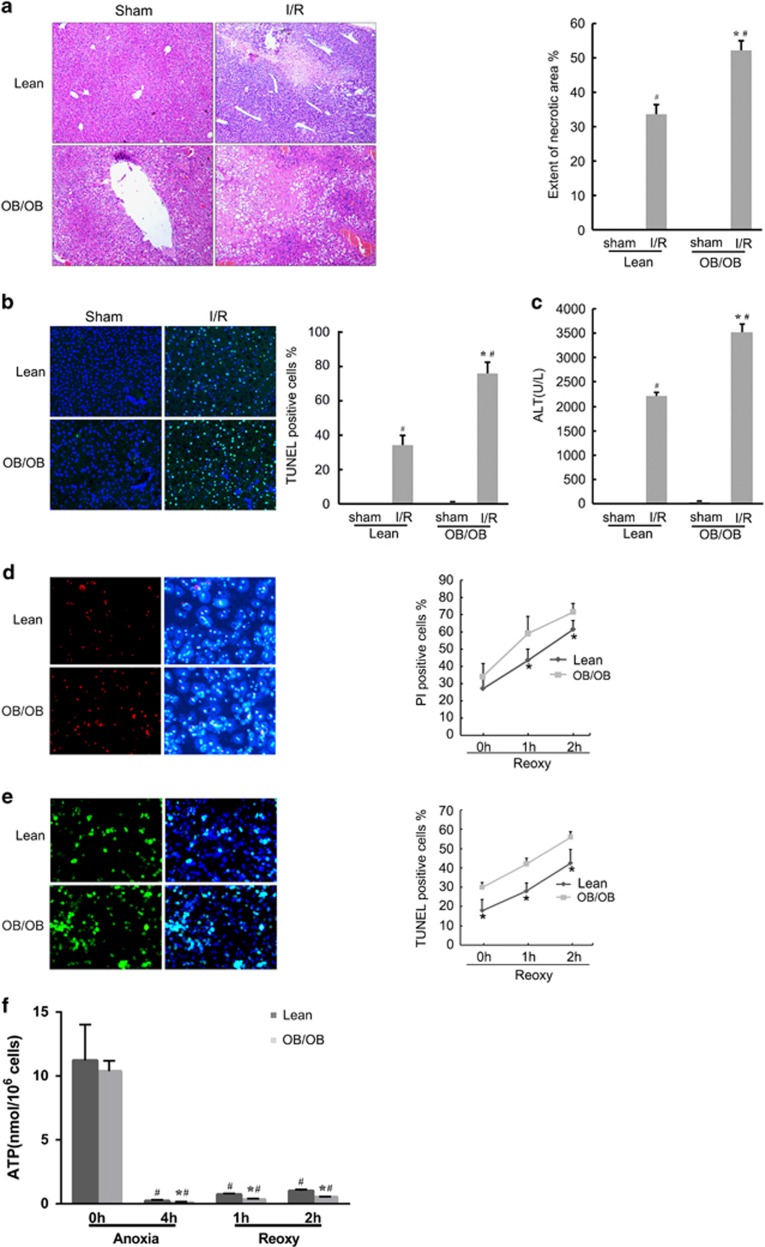
Fatty livers are more sensitive to I/R injury, both *in vivo* and *in vitro*. (**a**) Lean and ob/ob mice subjected to 1 h of liver ischemia and 24 h of reperfusion or sham operated. Liver histology images of the area of focal necrosis are shown (magnification × 200). Quantification of the necrotic areas is shown (right; *n*=4 per group). (**b**) After 24 h of reperfusion, TUNEL-positive cells were detected in lean livers and significantly increased in ob/ob mice livers. (**c**) Serum was obtained at 6 h of reperfusion and ALT levels were determined. (**d** and **e**) Isolated hepatocytes from lean and ob/ob mice underwent 4 h of anoxia and 2 h of reoxygenation. PI (red spots, **d**) and TUNEL (green spots, **e**) positive cells were calculated at each time point of reoxygenation. Cell nuclei were stained by Hoechst 33342 (blue spots). (**f**) Hepatocellular ATP concentration was determined at the indicated time points. ^#^*P*<0.05 *versus* the sham or 0 h of anoxia, *P<0.05 *versus* the lean group

The ob/ob mice were used as fatty liver model and wild-type C57BL/6 lean mice were used as controls in this study. The liver tissue was obtained at 1 h of ischemia and 24 h of reperfusion. The extent of necrotic areas was markedly increased in the fatty livers (Figure 2a). TdT-mediated dUTP nick end labeling (TUNEL) staining also showed that the apoptotic rate of hepatocytes in ob/ob mice was higher than in C57BL/6 mice (72.9±1.9% *versus* 30.1±1.2%, *P*<0.05; Figure 2b). The serum was obtained at 6 h of reperfusion. Liver injury marker alanine aminotransferase (ALT) in ob/ob mice was 1.6 times higher than in the normal controls (*P*<0.05; Figure 2c). The serum pro-inflammatory cytokines interleukin-6 (IL-6) and TNF-*α* levels were also increased in the ob/ob group (Supplementary Figures 2a and b).

In the hepatocyte anoxia/reoxygenation (A/R) *in vitro* model, increased necrosis (71.5±5.0% *versus* 61.5±5.0%, *P*<0.05) and apoptosis (56.0±2.8% *versus* 42.5±7.1%, *P*<0.05) were detected in steatotic hepatocytes, compared with the lean controls, after 2 h of reoxygenation (Figures 2d and e). The hepatocellular ATP were lower in steatotic hepatocytes both during 4 h of anoxia (0.11±0.05 *versus* 0.23±0.07 mmol/10^6^ cells, *P*<0.05) and 2 h of reoxygenation (0.38±0.08 *versus* 0.54±0.20 mmol/10^6^ cells, *P*<0.05), indicating less energy stored in steatotic hepatocytes during A/R (Figure 2f). Collectively, the fatty livers are more susceptible to I/R injury than the non-fatty livers.

### Low autophagic flux in steatotic hepatocytes during I/R injury

**Figure 3:**
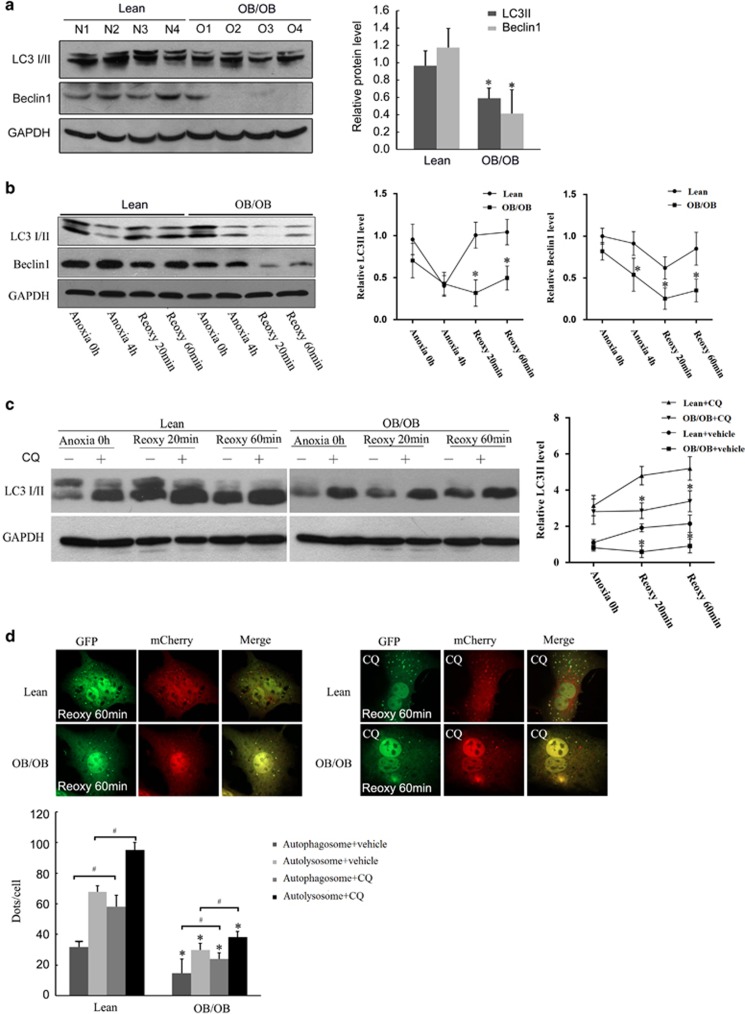
Decreased autophagic flux in steatotic hepatocytes during I/R. (**a**) Lean and ob/ob mice were subjected to 1-h ischemia and 6-h reperfusion. Liver LC3 and Beclin-1 expression was analyzed by western blotting (*n*=4 per group). Quantification of the relative protein levels normalized with GAPDH is shown at right. (**b**) Isolated hepatocytes from lean and ob/ob mice underwent 4 h of anoxia and 60 min of reoxygenation. The expression of LC3 and Beclin-1 was determined at the indicated time points. Quantification of LC3-II and Beclin-1 normalized with GAPDH are shown (right; *n*=4 per group). (**c**) Lean and steatotic hepatocytes pretreated with CQ (10 *μ*M) or vehicle controls for 1 h then subjected to 4 h of anoxia and 60 min of reoxygenation. LC3 expression was determined by western blotting at each time point. Densitometric analysis of LC3-II normalized with GAPDH is shown (right; *n*=4 per group). (**d**) Lean and steatotic hepatocytes infected with AdmCherry-GFP-LC3 were subjected to 4 h of anoxia and 60 min of reoxygenation. Confocal images of yellow (autophagosomes) and red (autolysosomes) puncta were collected with (right) and without (left) CQ (10 *μ*M) pretreatment. Quantification of the autophagosomes and autolysosomes at each group are shown (*n*=4 per group). ^#^*P*<0.05 *versus* no CQ treatment group, *P<0.05 *versus* the lean group

To investigate the effects of steatosis on hepatocellular autophagy during I/R, we examined liver LC3 and Beclin-1 expression by western blotting at 6 h of reperfusion. Two forms of LC3, LC3-I and LC3-II, are produced posttranslationally. LC3-I is cytosolic, whereas LC3-II is membrane bound and shows enrichment on the autophagosome vacuoles; for that reason, it is a useful indicator of autophagosomes. After 1 h of ischemia and 6 h of reperfusion, both LC3-II and Beclin-1 levels were reduced in the fatty liver (Figure 3a). Similarly, in the A/R model, the expression of LC3-II and Beclin-1 were significantly decreased, compared with the normal control samples, at both 20 and 60 min of reoxygenation in steatotic hepatocytes (Figure 3b).

To evaluate autophagic flux, we added chloroquine (CQ, 10 *μ*M), a lysosomal protease inhibitor, 1 h before A/R to inhibit autophagic flux. Immunoblotting showed that the constitutive autophagy turnover in the steatotic hepatocytes was comparable with lean hepatocytes. Reoxygenation of lean hepatocytes with CQ caused significantly increased LC3-II levels, indicating a rapid autophagic flux. However, low increase in expression of LC3-II was found in the steatotic hepatocytes with CQ treatment at reoxygenation, indicating a low autophagic flux (Figure 3c).

In order to better understand the autophagic flux, the hepatocytes were infected with Ad-mCherry-GFP-LC3. The immunofluorescent images of yellow (autophagosomes) and red (autolysosomes) puncta were counted after 4 h of anoxia and a subsequent 60 min of reoxygenation. Significant decrease of both autophagosomes and autolysosomes were found in steatotic hepatocytes (Figure 3d, left). CQ (10 *μ*M) treatment at 1 h before A/R significantly increased yellow puncta in lean hepatocytes, indicating a strong autophagic flux response to reoxygenation. However, in steatotic hepatocytes, the extent of CQ-induced accumulation of autophagosomes was significantly less than in normal cells. Together, these results demonstrate a reduced autophagic flux in steatotic hepatocytes during A/R.

### Autophagy regulates I/R injury in the fatty livers

**Figure 4:**
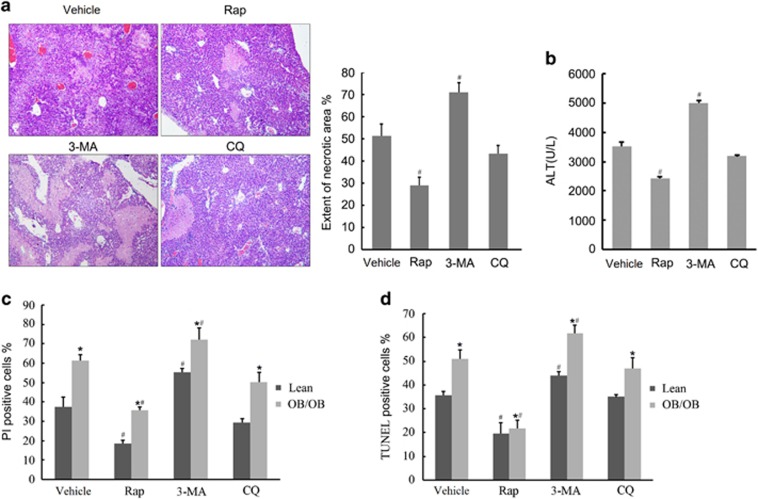
Autophagy regulates I/R injury in fatty livers. (**a**) Ob/ob mice were intraperitoneally injected with vehicle DMSO, rapamycin (Rap, 1 mg/kg), 3-MA (30 mg/kg) or CQ (60 mg/kg), respectively. Six hours after injection, mice were subjected to 1-h ischemia and 24-h reperfusion. Liver samples of ob/ob mice were obtained after 24 h of reperfusion. Necrotic area of each group was determined under light microscope (magnification × 200). Quantification of the necrotic areas is shown (right; *n*=5 per group). (**b**) Serum of ob/ob mice was obtained at 6 h of reperfusion and ALT levels were determined. (**c** and **d**) Isolated hepatocytes from lean and ob/ob mice pretreated with vehicle, rapamycin (0.2 *μ*M), 3-MA (5 mM) or CQ (10 *μ*M) for 1 h, respectively, and then underwent 4-h anoxia and 2 h of reoxygenation. Densitometric analysis of PI (**c**) and TUNEL (**d**) assay is shown. ^#^*P*<0.05 *versus* vehicle controls, *P<0.05 *versus* the lean group

To investigate the impact of autophagy on fatty liver I/R injury, 6 h before I/R, we treated the ob/ob mice with rapamycin (1 mg/kg), an inhibitor of mTOR, to stimulate autophagy, and 3-methyladenine (3-MA, 30 mg/kg), an inhibitor of type III phosphatidylinositol 3-kinases (PI-3K), to suppress autophagy, or CQ (60 mg/kg), respectively. Rapamycin significantly reduced the areas of liver necrosis and serum ALT levels in response to reperfusion injury, compared with vehicle controls (Figures 4a and b). The protective effects were also demonstrated by diminished IL-6 and TNF-*α* production after 6 h of reperfusion (Supplementary Figures 3a and b). In hepatocyte A/R experiments, propidium iodide (PI) and TUNEL assay after 4 h of anoxia and 2 h of reoxygenation also displayed decreased cell death and apoptosis in the rapamycin (0.2 *μ*M) pretreatment group in steatotic hepatocytes (Figures 4c and d). Conversely, autophagy inhibitor 3-MA (5 mM) resulted in aggravating I/R injury and no significant liver damage change was found in the CQ treatment group, both *in vivo* and *in vitro*. These results indicate that autophagy has an active role in regulating I/R injury of fatty livers.

### Calpain 2 activation aggravates I/R injury in fatty livers

**Figure 5:**
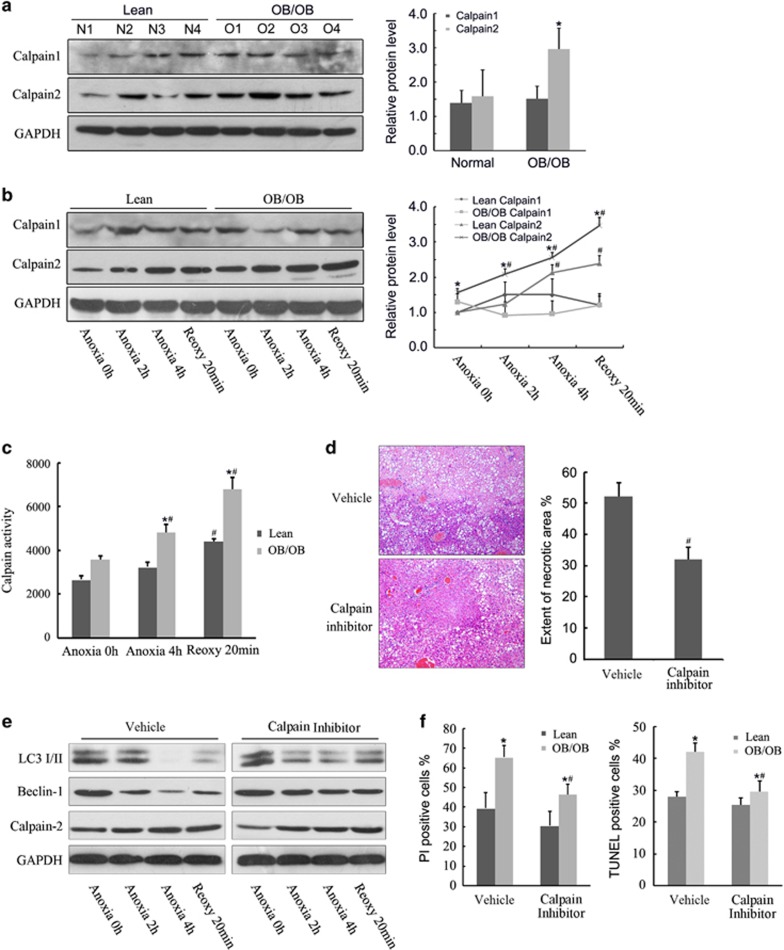
Calpain 2 is activated and aggravates I/R injury in fatty livers. (**a**) Lean and ob/ob mice were subjected to 1 h of ischemia and 6 h of reperfusion. Liver calpain 1 and calpain 2 expression levels were analyzed by western blotting. Quantification of the relative protein level is shown (right; *n*=4 per group). (**b**) Isolated hepatocytes from lean and ob/ob mice underwent 4 h of anoxia and 20 min of reoxygenation. Calpain 1 and calpain 2 expression levels were determined at each time point. Quantification of relative protein level is shown (right; *n*=4 per group). (**c**) Calpain activity was determined by SLLVY-AMC fluorometry at each time point. (**d**). Ob/ob mice were pretreated with calpain inhibitor III (10 mg/kg) or vehicle DMSO for 6 h and then subjected to 1-h ischemia and 24 h of reperfusion. Necrotic area of liver samples was identified under light microscope (200x). Quantification of the necrotic areas is shown (right; *n*=4). (**e** and **f**) Isolated hepatocytes from ob/ob mice pretreated with calpain inhibitor III (25 *μ*M) or vehicle for 1 h, and then subjected to 4 h of anoxia and 20 min of reoxygenation. Changes in LC3, Beclin-1 and calpain 2 were determined by western blotting at each time points (**e**). Densitometric analysis of PI and TUNEL assay at 20 min of reoxygenation is shown (**f**). ^#^*P*<0.05 *versus* anoxia 0 h or vehicle controls, *P<0.05 *versus* the lean group

Calpains are upregulated in steatosis and hydrolyze autophagy proteins.^12^ To investigate the potential involvement of calpains in autophagy protein depletion, calpains expression and activity were determined. Immunoblotting showed higher expression of calpain 2 but not calpain 1 in the fatty liver group after 6 h of reperfusion (Figure 5a). In steatotic hepatocytes, calpain 2 expression was increased, whereas no significant change was found in calpain 1 expression during A/R (Figure 5b). Calpain activity was also significantly enhanced in steatotic hepatocytes compared with lean controls during A/R (Figure 5c). Calpain inhibition by calpain inhibitor III (10 mg/kg) pretreatment protected the fatty livers from I/R injury as demonstrated by decreased hepatocellular necrotic areas, serum ALT and pro-inflammatory cytokine levels after reperfusion (Figure 5d and Supplementary Figures 4a–c). In steatotic hepatocyte A/R experiments, calpain inhibitor III (25 *μ*M) pretreatment reversed LC3-II and Beclin-1 downregulation during A/R (Figure 5e). Notably, calpain inhibitor III successfully reduced A/R injury of the steatotic hepatocytes, but lost its protective effects in lean control hepatocytes (Figure 5f). Thus, these results show an integral role of calpain 2 in I/R injury to fatty livers.

### Cleavage of Atg3 and Atg7 by calpain 2 during fatty liver I/R

**Figure 6:**
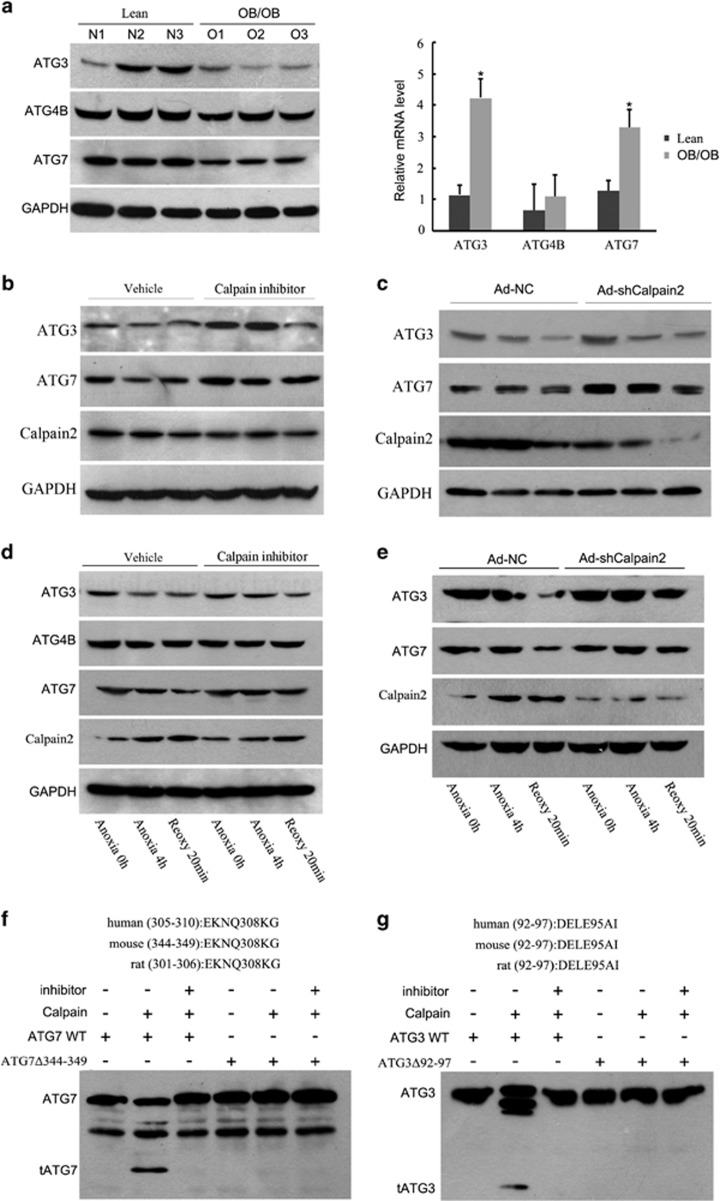
Calpain 2 degrades Atg3 and Atg7. (**a**) Lean and ob/ob mice were subjected to 1 h of ischemia and 6 h of reperfusion. The expression of liver autophagy-related proteins Atg3, Atg4B and Atg7 were compared by western blotting (left, *n*=3). The abundance of Atg3, Atg4B and Atg7 mRNAs were determined by RT-qPCR (right, *n*=3). (**b** and **c**) Ob/ob mice were pretreated with calpain inhibitor III (10 mg/kg) or vehicle DMSO for 6 h (**b**), or ob/ob mice were transduced with Ad-shCalpain2 or control virus (Ad-NC; 2 × 10^11^vp/mice) for 7 days, respectively (**c**), and then subjected to 1-h ischemia and 6 h of reperfusion. Liver Atg3, Atg7 and calpain 2 expression levels at each group were determined by western blotting. Quantification of relative protein level is shown in Supplementary Figures 6a and b. (**d**) Hepatocytes isolated from ob/ob mice were subjected to 4 h of anoxia and 20 min of reoxygenation in the presence or absence of calpain inhibitor III (25 *μ*M) pretreatment for 1 h. Atg3, Atg4B, Atg7 and Calpian 2 expression levels were determined by western blotting at indicated time points. (**e**) Hepatocytes isolated from ob/ob mice infected with Ad-shCalpain2 or control virus (Ad-NC) for 48 h and then subjected to 4 h of anoxia and 20 min of reoxygenation. Western blotting of the relative protein level is shown. Quantification of the relative protein level of (**d**) and (**e**) is shown in Supplementary Figures 6c and d. (**f** and **g**) For the *in vitro* calpain cleavage assay, wild-type Atg7 or Atg7 mutant at the 344–349 amino acid (**f**), Atg3 or Atg3 mutant at the 92–97 amino acid (**g**) recombinant proteins that translated by TnT Translation System, were incubated with recombinant calpain in the presence or absence of 25 *μ*M calpain inhibitor III at 30 °C for 10 min. Then the reaction was terminated and subjected to western blot. *P<0.05 *versus* the lean group

We then explored the mechanism underlying diminished autophagy in fatty liver I/R. The mRNA expression patterns of Atgs were examined in both ob/ob and normal mice livers after 6 h of reperfusion. Surprisingly, there were increased, or at least no decrease, in autophagy-related gene mRNA levels in ob/ob mice (Supplementary Figure 5a). We then tested the protein expression levels of autophagy-related genes (Supplementary Figure 5b). The Atg3 and Atg7 protein levels were markedly decreased in the fatty livers after 6 h of reperfusion (Figure 6a, left). The corresponding mRNA levels were remarkably elevated (Figure 6a, right), indicating that the Atg3 and Atg7 proteins may be degraded during the course of reperfusion.

We then found calpain inhibitor III (10 mg/kg) pretreatment or Ad-shcalpain2 (2 × 10^11^vp/mice) infection could reverse the reduction of both Atg3 and Atg7 in fatty livers at 6 h of reperfusion (Figures 6b and c and Supplementary Figures 6a and b). We next confirmed these results in hepatocyte A/R experiments (Figures 6d and e and Supplementary Figures 6c and d). *In vitro* cleavage experiments showed direct cleavage of Atg7 by calpain 2, whereas pretreatment with calpain inhibitor III or deletion of the cleavage site at amino acids 344–349 in Atg7 reversed this cleavage (Figure 6f). Similar results were found with Atg3, and loss of amino acids 92–97 led to resistance to calpain 2-mediated degradation (Figure 6g). Taken together, these results suggest that calpain 2 activation contribute to Atg3 and Atg7 depletion in fatty liver I/R injury.

### Overexpression of Atg3 or Atg7 rescues autophagy and protects the fatty liver from I/R injury

**Figure 7:**
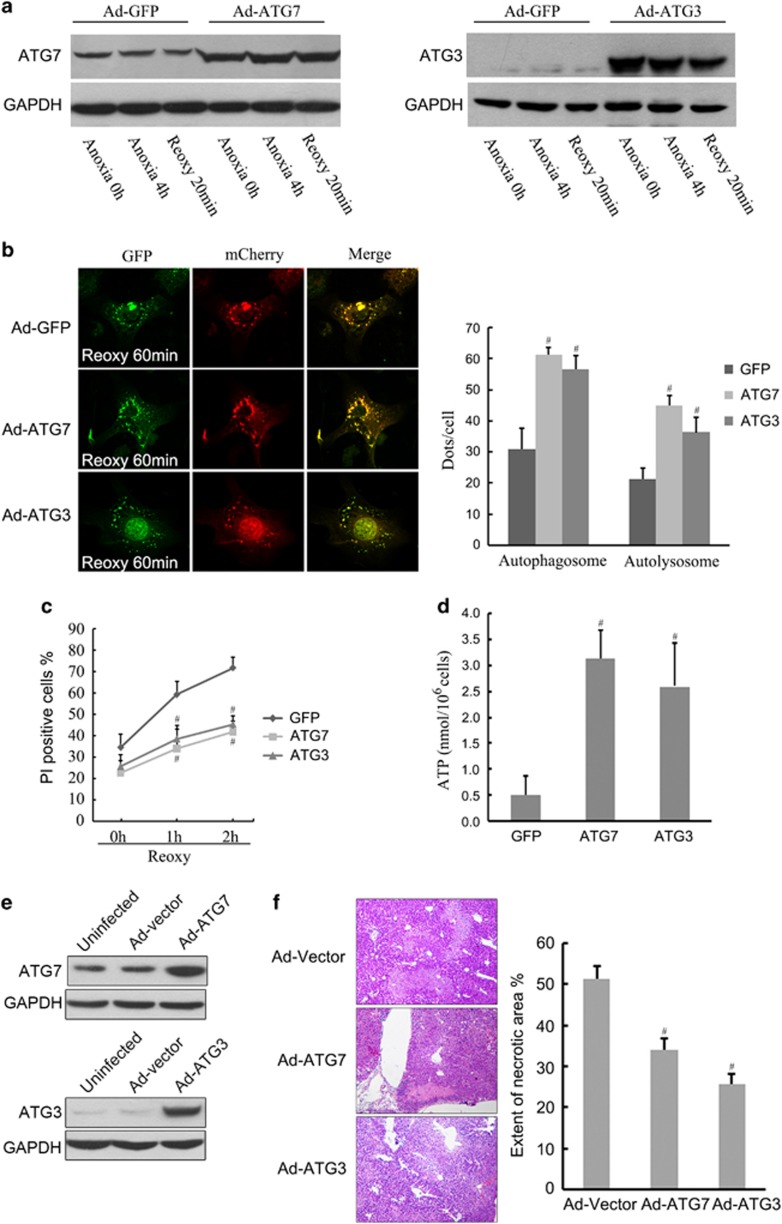
Overexpression of Atg3 or Atg7 rescues autophagy and protects steatotic hepatocytes from A/R injury. (**a**) Hepatocytes isolated from ob/ob mice were infected with AdAtg7 or AdAtg3 (10 p.f.u./cell) for 48 h and then subjected to 4-h anoxia and 20 min of reoxygenation. AdGFP was used for infection control. Western blotting analysis of Atg7 and Atg3 was performed at indicated time points. (**b**) Isolated ob/ob hepatocytes were infected with AdmCherry-GFP-LC3/AdGFP, AdmCherry-GFP-LC3/AdAtg7 or AdmCherry-GFP-LC3/AdAtg3 (10 p.f.u./cell), respectively, for 48 h, and then subjected to 4 h of anoxia and 60 min of reoxygenation. Confocal images of hepatocytes were collected. Quantification of autophagosomes and autolysosomes are shown at right. (**c**) PI-positive cells were identified by fluorometry at indicated time points of reoxygenation. (**d**) Hepatocellular ATP concentration was determined by luciferin/luciferase ATP assay after 60 min of reoxygenation. (**e** and **f**) Ob/ob mice were transduced with Advector, AdAtg7 or AdAtg3 (2 × 10^11^vp/mice) for 7 days respectively, and then subjected to 1 h of ischemia and 24 h of reperfusion. Liver Atg3 and Atg7 expression levels at each group were determined by western blotting (**e**). Liver necrotic areas were determined under light microscope (200x). Quantification of the necrotic areas are shown (right; *n*=4) (**f**). ^#^*P*<0.05 *versus* AdGFP or Advector

To investigate the roles of Atg3 and Atg7 in A/R injury, steatotic hepatocytes were infected with AdAtg3 or AdAtg7 before A/R (Figure 7a). Autophagy levels were elevated as evidenced by increased yellow puncta (autophagosomes) and red puncta (autolysosomes) in steatotic hepatocytes after reoxygenation (Figure 7b). Overexpression of Atg3 or Atg7 resulted in elevation of cellular ATP levels and decreased cell death after reoxygenation (Figures 7c and d).

To further validate our findings with *in vivo* I/R, ob/ob mice were transduced with AdAtg3 or AdAtg7 and subjected to 1 h of ischemia and 24 h of reperfusion. Consistent with the *in vitro* data, successful overexpression of Atg3 or Atg7 suppressed reperfusion induced cell death and pro-inflammatory cytokines production (Figures 7e and f and Supplementary Figures 7a–c). These results suggest a causative role of Atg3 and Atg7 in autophagy deficiency and subsequent I/R injury of fatty livers.

## Discussion

Liver steatosis is a complicated pathophysiological change involving dysfunction of multiple intracellular systems such as mitochondrial dysfunction, ROS generation and ER stress.^13^ Autophagy is usually required to relieve ROS damage by eliminating damaged mitochondria and to relieve ER stress by degrading misfolded proteins. However, hepatocyte lipid accumulation can decrease autophagy, and impaired autophagy further aggravates steatosis and inflammation.^6^ I/R is a vigorous challenge for fatty livers and a certain level of autophagy is needed for cell survival. This study provides the first evidence that the autophagic system in steatotic hepatocytes is fragile during I/R, demonstrated by marked decreased autophagosome and autolysosome formation during reperfusion. To exclude the possibility that impaired autophagy was caused by a decline in lysosomes, the lysosome marker lysosome-related membrane protein-2 was examined and no significant changes were found (Supplementary Figure 5c). Cumulatively, these results indicate that autophagy in steatotic hepatocytes is severely impaired during I/R.

The protective effect of autophagy during I/R has been demonstrated by numerous studies.^6^ Pharmacological activation of autophagy by rapamycin or chloramphenicol succinate can protect myocardial cells from I/R injury.^14, 15^ Overexpression of Beclin-1 promotes autophagic flux and prevents hepatocyte death during I/R.^16^ Fasting is reported to protect the liver from I/R injury by induction of autophagy.^17^ However, under prolonged I/R injury conditions, overactivated autophagy may not benefit cell survival. The reason is that autophagosomes form and engulf targets, but cannot fuse with lysosomes and clear their contents. The cell may respond by ejecting the autophagosomes from the cell, thereby eliciting an acute and significant inflammatory response. In this setting, suppressed autophagy may be preferable.^18^ In our study, we clearly demonstrated that there is low autophagosome formation and no significant impairment of autophagosome and lysosome fusion in fatty liver I/R. Inducing autophagy, whether by pharmacological agents or by gene overexpression, significantly protects cell from I/R injury. We noticed CQ did not affect the liver damage as the autophagy inhibitor. The main reason is CQ has a dual effect in liver I/R injury. For one hand, CQ could aggravate liver I/R injury through inhibition of autophagy, but on the other hand it could protect the liver against I/R injury via modulating mitogen-activated protein kinase activation, reducing high-mobility group box 1 release and inflammatory cytokines production.^19^ It has been reported that ischemia preconditioning induces autophagy and limits necrosis in human recipients of fatty liver grafts.^20^ Moreover, melatonin and trimetazidine cocktail improves fatty liver allograft preservation through increasing autophagy and reducing ER stress.^21^ Thus, our results are consistent with previous research and demonstrate a crucial role for autophagy in fatty liver I/R injury.

Defective autophagosome formation is usually associated with downregulation of Atg protein. Compared with lean control liver at reperfusion, we found significantly increased mRNA of *Atg1, Atg3, Atg5, Atg7 and Atg12* (Supplementary Figure 5a), which are reported to be transcriptionally regulated by farnesoid X receptor (FXR).^22^ Downregulation of FXR is found in fatty liver and may contribute to *Atg* mRNA elevation, which may be a compensative response to autophagy proteins depletion.^23^ After screening of Atg expression, we only found significant downregulation of Atg3 and Atg7 in fatty liver. A close interaction exists between Atg3 and Atg7, both of which are involved in and essential to autophagy elongation. First, Atg7 and Atg3 are needed during Atg5-Atg12 conjugation. In addition, LC3-I is conjugated to phosphatidylethanolamine in a reaction involving Atg7 and then Atg3 to form LC3-II.^24^ However, our study identified that overexpression of either Atg3 or Atg7 successfully increased autophagy activity in steatotic hepatocytes during I/R. This suggests that Atg3 and Atg7 may be involved in an independent pathway in autophagy elongation, although the exact mechanism needs further investigation.

There is evidence to imply that Atg3 and Atg7 are the targets of calpain systems. It has been reported that Atg7 can be cleaved by calpain 2 in fatty livers.^12^ Furthermore, during hepatocyte anoxia and reoxygenation, Atg7 may also be degraded by calpain 2.^25^ *In vitro* studies have identified that Atg3 can be cleaved by calpains.^26^ Moreover, calpain activity was found to be increased in steatotic hepatocytes.^12^ We found that the expression of calpain 2, but not calpain 1 or caspases, was markedly increased in fatty livers during I/R. Inhibition of calpain 2 significantly restored Atg3 and Atg7 and reduced I/R injury in ob/ob mice. Calpain 2 is activated by Ca^2+^ in the cytoplasm. Pharmacological inhibition of calcium channels using the FDA-approved drug verapamil was reported to successfully restore autophagic flux and reduce steatohepatitis.^27^ As calcium channel blockers have been safely used clinically for >30 years for the treatment of hypertension, whether it can suppress calpain 2 activity and reduce fatty liver I/R injury would be an interesting clinical topic. It is nevertheless possible that events other than calpain 2 activation may be attributed to Atg3 and Atg7 reduction. For instance, steatotic tissues manifest higher basal levels of mTOR complex 1 (mTORC1) than normal tissues, and I/R further increases mTORC1 activity. It is also known that mTORC1 can suppress Atg3 and Atg7.^28^ Thus, combined use of calpain inhibitors and mTOR inhibitors may induce a synergistic effect in protecting against fatty liver I/R injury.

In conclusion, this article demonstrates that impaired autophagy is one of the key reasons for increased sensitivity of fatty livers to I/R injury. Calpain 2-mediated cleavage of Atg3 and Atg7 accounts for the impaired autophagy. Targeting Atg3, Atg7 and calpain 2 could be a novel strategy to improve liver function in patients with fatty liver resection or transplantation.

## Materials and Methods

### Patients and tissues

Biopsy samples including steatotic (*n*=23) and normal control (*n*=23) liver tissues were obtained from the liver allografts of 46 patients who had undergone liver transplantation at The First Affiliated Hospital of Sun Yat-sen University (Guangzhou, China) between 2012 and 2015. The samples were obtained 1 h after hepatic artery reperfusion. Ethical approval for research on human subjects was obtained from the Institutional Review Board of The First Affiliated Hospital of Sun Yat-sen University, and written informed consents were obtained from each patient.

### IHC staining

Liver tissues were embedded in paraffin and cut into slices. Each slice was deparaffinized using xylene and an ethanol gradient. Antigen retrieval was performed by boiling in 10 mM citrate buffer for 10 min and cooling for 20–30 min. Endogenous peroxidase activity was quenched with 3% H_2_O_2_ for 10 min, and the slices then were washed three times in PBS for 2 min. Blocking of nonspecific binding was performed with 1% goat serum. The samples were incubated at 4 °C overnight with a 1 : 50 dilution of anti-LC3 or Beclin-1 antibody (Cell Signaling Technology, Danvers, MA, USA). The samples were incubated with a biotin-conjugated goat anti-rabbit secondary antibody conjugated to biotin (Vector Laboratories, Burlingame, CA, USA) at a dilution of 1 : 200 for 30 min. The sections were then stained with a VECTASTAIN ABC Kit (Vector Laboratories) according to the manufacturer's instructions. Staining was detected with a substrate solution of diaminobenzidine tetrahydrochloride (Sigma-Aldrich, Milan, Italy). Slices were then subjected to hematoxylin staining for 45 s. After dehydration with an ethanol gradient and treatment with xylene, slices were mounted in mounting medium (DAKO, Glostrup, Denmark).

Each section was imaged on a BX51WI microscope (Olympus, Tokyo, Japan). Staining scores were calculated with the following method as described previously.^29^ Positive cell level was graded as 0–IV (0, no positive cells; I, <25% positive cells; II, 25–50% positive cells; III, 50–75% positive cells; IV, >75% positive cells). The intensity of positive staining was graded as I–III (I, weak yellow staining; II, strong and brown staining; III, very strong and deep brown staining). We then summed the proportion and intensity grade level to obtain a total grade level (range, 0–VII). According to the calculated level, the staining level was scored using three-point scale: grade level of 0, scored 0; grades level of I–IV, score 1; grades level of 5 and more, score 2.

### Animals

Male C57BL/6J wild-type mice and C57BL/6J-ob/ob mice (all 10–12 weeks of age) were purchased from Model Animal Research Center of Nanjing University. All mice were housed in an environment with controlled light (12-h light/dark), humidity, and temperature, with food and water available. All animal protocols were approved by the Animal Care Committee of Sun Yat-sen University.

### Hepatic I/R injury model

Mice were anesthetized with ketamine (60 mg/kg, i.p.) and xylazine (8 mg/kg, i.p.). After laparotomy, an atraumatic clip (Fine Science Tools, North Vancouver, BC, Canada) was used to interrupt the arterial and portal venous blood supply to the left and middle liver lobes. After 1 h of partial warm ischemia, reperfusion was initiated by clamp removal. Blood and ischemic liver tissues were removed, frozen in liquid nitrogen and kept at −80 °C until processing. Sham controls were prepared according to the same procedure, but without vascular occlusion.

For calpain inhibition experiments, male ob/ob mice were injected with calpain inhibitor III (Calbiochem, La Jolla, CA, USA) at 10 mg/kg or vehicle (dimethylsulfoxide, DMSO) via intraperitoneal injection. For autophagy activation or inhibition, rapamycin (at 1 mg/kg, Sigma-Aldrich), 3-MA (at 30 mg/kg, Sigma-Aldrich), CQ (at 60 mg/kg, Sigma-Aldrich) were injected. Six hours after injection, mice underwent I/R and blood and liver tissues were obtained for analysis.

### Liver histological examination

Liver sections stained with hematoxylin–eosin were used for histological determination of liver injury. Steatosis was assessed as outlined by Kleiner *et al.*^30^ The percentage of necrosis was calculated by assessing the area of necrosis compared with the entire histological section.

### Hepatocyte isolation and culture

Hepatocytes were isolated from mice by the collagenase perfusion method, as previously described.^31^ The cells were then suspended in high glucose Dulbecco's modified Eagle's medium (DMEM) supplemented with 10% (v/v) fetal bovine serum, 1% (v/v) penicillin/streptomycin, 1% (v/v) nonessential amino acids, dexamethasone 10^−7^ mol/l and insulin 10^−6^ mol/l. The cells were plated onto gel-coated plates (5 × 10^6^ cells per well) and allowed to attach to the plates. After 2 h, the cells were washed twice with PBS and the media replaced with high glucose DMEM supplemented with 1% (v/v) penicillin/streptomycin, 0.5% (v/v) gentamycin (10 mg/ml), 1% (v/v) nonessential amino acids, dexamethasone 10^−7^ mol/l and insulin 10^−6^ mol/l. The cells were subsequently cultured overnight.

### Anoxia/reoxygenation

For anoxia, hepatocytes were incubated for 4 h at 37 °C in Krebs-Ringer-hydroxyethylpiperazine-N-2 ethanesulfonic acid (KRH) buffer (pH 6.2) in an anaerobic chamber (Thermo Fisher Scientific Inc., Waltham, MA, USA). To simulate reoxygenation, the medium was replaced with aerobic KRH at pH 7.4. For mechanistic study, calpain inhibitor III (25 *μ*M), rapamycin (0.2 *μ*M), 3-MA (5 mM) or CQ (10 *μ*M) were applied as indicated.

### Western blotting analysis

Liver tissue was homogenized and primary cultured hepatocytes were lysed in RIPA buffer containing protease and phosphatase inhibitors. Protein concentration was assessed with a Bradford assay and equal amounts of total protein were separated by SDS-PAGE electrophoresis, transferred to PVDF membranes and blocked with 5% skimmed milk powder for 1 h at room temperature. The PVDF membranes were washed with TBST (containing NaCl, Tris-Hcl and Tween-20) and incubated overnight with primary antibodies against LC3, Beclin-1, calpain 1, calpain 2, Atg7, GAPDH (Cell Signaling Technology), Atg3 and Atg4B (Abcam, Cambridge, MA, USA) at 4 °C. The following day, the membranes were washed twice with TBST. Membranes were incubated with the appropriate secondary antibodies for 1 h at room temperature and washed three times with TBST. The bound proteins were visualized by chemiluminescence (ECL, Forevergen, Guangzhou, China).

### Circulating levels of transaminases

Serum ALT activity was measured with a commercially available test kit (Biovision, Milpitas, CA, USA), according to the manufacturer's instructions.

### Cytokine measurement

Serum of C57BL/6J wild-type mice and ob/ob mice was obtained after 1 h of ischemia and 6 h of reperfusion. IL-6 and TNF-*α* were measured with mouse IL-6 ELISA kit (Biovision) and TNF-*α* ELISA Kit (Cusabio, Wuhan, China).

### Necrosis and apoptosis assay

Necrosis and apoptosis were assessed by PI fluorometry and TUNEL staining using an In Situ Apoptosis Kit (Roche, Penzberg, Germany). Specimens were then evaluated by light microscopy.

### Real-time reverse transcription-PCR (RT-qPCR)

Total RNA was isolated from tissue samples with TRIzol (Invitrogen, Carlsbad, CA, USA) according to the manufacturer's instructions. RNA was reverse transcribed by ReverTra Ace qPCR RT Kit (Toyobo Biochemicals, Osaka, Japan) according to the manufacturer's protocol. Real-time PCR was performed with GoTaq qPCR Master Mix (Promega, Madison, WI, USA) on a MiniOpticon Real-Time PCR detection instrument (Bio-Rad, Hercules, CA, USA) using the SyBr Green detection protocol as outlined by the manufacturer. Briefly, the amplification mixture consisted of 0.5 *μ*M primers, 25 *μ*l of GoTaq qPCR Master Mix, and 1 *μ*l template DNA in a total volume of 40 *μ*l. Samples were amplified with the following program: initial denaturation at 98 °C for 30 s, followed by 40 cycles of denaturation for 15 s at 98 °C and annealing/elongation for 60 s at 60 °C. All PCR amplification processes were run in triplicate, and control reactions without template were included. RNA expression change was calculated relative to the control using the ΔΔCt method. Data shown represent the mean ±S.E. of three separate experiments. Sequence-specific primers are presented in Supplementary Methods.

### Adenovirus construction and transfection

Recombinant adenoviruses (Ads) containing Atg7, Atg3, or mCherry-GFP-LC3 were generated according to the protocol described for the AdEasy XL Ad Vector System (Stratagene, La Jolla, CA, USA). Hepatocytes were infected at the concentration of 10 p.f.u./cell for 48 h then subjected to A/R. AdGFP was used for infection control. Images of AdmCherry-GFP-LC3 were captured on a Delta-Vision RT Imaging Microscope System (Applied Precision, Bratislava, Slovakia). For *in vivo* study, adenovirus was delivered into mice via the orbital venous plexus at a titer of 2 × 10^11^vp/mice. After 7 days, mice were subjected to I/R.

### Measurement of calpain activity

A fluorogenic-substrate assay, using a membrane permeable calpain substrate, succinyl-Leu-Leu-Val-Tyr-7-amino-4-methylcoumarin (SLLVY-AMC; Sigma, St. Louis, MO, USA), was used to detect calpain activity in cell extracts in the presence and absence of calpain inhibitor III. Cell lysates were centrifuged (15 000 × *g* for 5 min) and the supernatants were used for the assay: 40 *μ*g of total protein were diluted to 50 *μ*l with calpain reaction buffer (50 mM Tris-HCl pH 7.5, 10 mM CaCl2, 30 mMNaCl, 5 mM DTT) containing 100 *μ*M Suc-LLVY-AMC. Production of fluorescent AMC was monitored continuously (excitation 380 nm and emission 460 nm) by fluorescent plate reader.

### ATP measurement

ATP levels in cultured hepatocytes were determined by a luciferin/luciferase ATP assay kit (Promega) according to the manufacturer's instructions. Cells were washed with PBS and harvested under normal culture conditions. Total ATP was extracted in lysis buffer containing 100 mM potassium phosphate buffer (pH 7.8), 2 mM EDTA, 1 mM DTT and 1% Triton X-100. The ATP level in each cell lysate was determined by bioluminescent luciferase assay and emitted light was measured by luminometer.

### Calpain cleavage assay

The mice Atg7 wild-type, Atg7 mutant with the 344–349 amino-acid deletion (Δ344–349), Atg3 wild-type, and Atg3 mutant with the 92–97 amino-acid deletion (Δ92–97) cDNA were cloned into the pcDNA3.1 expression vector, and recombinant proteins were translated using a TnT Quick Coupled Transcription/Translation System (Promega) according to the manufacturer's protocol. For *in vitro* calpain cleavage assays, TNT reaction products were incubated in calpain cleavage buffer (50 mM Tris-HCl pH 7.4, 50 mM NaCl, 1 mM EDTA, 1 mM EGTA and 1 mM DTT) containing 0.3 units/ml recombinant calpain (Calbiochem) and 5 mM CaCl_2_. Samples were then incubated at 30 °C for 10 min in the presence or absence of 25 *μ*M calpain inhibitor III. The reactions were terminated by the addition of 3 × Laemmli buffer followed by boiling for 5 min. The samples were then subjected to western blot.

### Statistical analysis

Values are presented as mean±S.D. The correlation between LC3 expression and degree of steatosis was evaluated by Pearson correlation analysis, performed with the GraphPad Prism software (GraphPad Software Inc., La Jolla, CA, USA). Kaplan–Meier curves were used to estimate patient survival among groups. The log-rank test was applied to assess a statistical difference between survival curves. Statistical analysis was performed using the Student's *t*-test or one-way analysis of variance (ANOVA) with SPSS software package for Windows (SPSS 15.0 for Windows; SPSS Inc., Chicago, IL, USA). *P*<0.05 was considered statistically significant. All experiments are representative of at least three different cell isolations or animals per group.

## Abbreviations

I/R: ischemia and reperfusion
ROS: reactive oxygen species
ER: endoplasmic reticulum
ATP: adenosine triphosphate
Atg: autophagy-related protein
PKB: protein kinase B
mTOR: mammalian target of rapamycin
TUNEL: TdT-mediated dUTP nick end labeling
ALT: alanine aminotransferase
IL-6: interleukin-6
TNF-*α*: tumor necrosis factor-*α*
A/R: anoxia/reoxygenation
3-MA: 3-methyladenine
PI-3K: type III phosphatidylinositol 3-kinases
CQ: chloroquine
PI: propidium iodide
mTORC1: mammalian target of rapamycin complex 1
