# Supplementary material for: Calpain 2-mediated autophagy defect increases susceptibility of fatty livers to ischemia–reperfusion injury
Source: Cell Death Dis. 2016 Apr 14;7(4):e2186–. doi: 10.1038/cddis.2016.66 (PMC4855654; doi:10.1038/cddis.2016.66)

**Calpain 2 mediated autophagy defect increases susceptibility of fatty liver to ischemia reperfusion injury**

Qiang Zhao, Zhiyong Guo, Wen Deng, Shunjun Fu, Chuanzhao Zhang, Maogen Chen, Weiqiang Ju, Dongping Wang, XiaoShun He

**Methods**

**Real-time reverse transcription-PCR (RT-qPCR).**

Sequence-specific primers were as follows:

1. ATG1

Forward primerAAGTTCGAGTTCTCTCGCAAG

Reverse primerCGATGTTTTCGTGCTTTAGTTCC

1. ATG3

Forward primerACACGGTGAAGGGAAAGGC

Reverse primerTGGTGGACTAAGTGATCTCCAG

1. ATG4B

Forward primerTATGATACTCTCCGGTTTGCTGA

Reverse primerGTTCCCCCAATAGCTGGAAAG

1. ATG5

Forward primerTGTGCTTCGAGATGTGTGGTT

Reverse primerGTCAAATAGCTGACTCTTGGCAA

1. ATG6

Forward primerATGGAGGGGTCTAAGGCGTC

Reverse primerTCCTCTCCTGAGTTAGCCTCT

1. ATG7

Forward primerGTTCGCCCCCTTTAATAGTGC

Reverse primerTGAACTCCAACGTCAAGCGG

1. ATG8

Forward primerTTATAGAGCGATACAAGGGGGAG

Reverse primerCGCCGTCTGATTATCTTGATGAG

1. ATG10

Forward primerGTAGTTACCAAGTGCCGGTTC

Reverse primerAGCTAACGGTCTCCCATCTAAA

1. ATG12

Forward primerAACAAAGAAATGGGCTGTGG

Reverse primerTTTGCAGTAATGCAGGACCA

1. p62

Forward primerGGCACCGGAGGGTTTAATTTT

Reverse primerGCAGGTGTTGTTCCAAAGTTG

1. Calpain2

Forward primerGGTCGCATGAGAGAGCCATC

Reverse primerCCCCGAGTTTTGCTGGAGTA

1. GAPDH

Forward primer AGGTCGGTGTGAACGGATTTG

Reverse primer TGTAGACCATGTAGTTGAGGTCA

**Table S1. General character of liver transplant recipient**

|  | **Fatty liver group (n=23)** | **Control group (n=23)** | **P value** |
| --- | --- | --- | --- |
| Age | 47.1±9.4 | 46.0±13.9 | 0.267 |
| Gender |  |  | 0.636 |
| Male | 21(91.3%) | 20(87.0%) |  |
| Diagnosis of recipients |  |  | 0.741 |
| HBV-related HCC | 11 | 11 |  |
| HBV-related liver cirrhosis | 9 | 7 |  |
| Fulminant hepatic failure | 2 | 2 |  |
| Others | 1 | 3 |  |
| Child-Pugh score | 9.0±1.9 | 10.0±1.7 | 0.528 |
| MELD score | 20.1±10.9 | 20.9±13.7 | 0.236 |

**Abbreviations:** HCC, hepatocellular carcinoma; HBV, hepatitis b virus; MELD, model of end-stage liver disease.

**Figure Legends**

**Figure S1.** Kaplan-Meier survival curves of overall survival in fatty liver group (n=23) according to the summed LC3 and Beclin-1 expression score. Low expression score: sum of LC3 and Beclin-1 score≤1；High expression score: sum of LC3 and Beclin-1 score＞1.

**Figure S2.** Lean and ob/ob mice subjected to one hour of liver ischemia and six hours of reperfusion or sham operated. Serum IL-6 (a) and TNF-α (b) levels were determined. #P＜0.05 versus the sham group, *P＜0.05 versus the lean group.

**Figure S3.** Ob/ob mice were intraperitoneally injected with vehicle DMSO, autophagy enhancer rapamycin (Rap, 1mg/kg), autophagy inhibitor 3-Methyladenine (3-MA, 30mg/kg) or autophagosome and autolysosome inhibitor chloroquine (CQ, 60mg/kg), respectively. Six hours after injection, mice were subjected to one hour ischemia and six hours reperfusion. Serum IL-6 (a) and TNF-α (b) levels were determined. #P＜0.05 versus vehicle control.

**Figure S4.** Ob/ob mice were pretreated with calpain inhibitor III (10mg/kg) or vehicle DMSO for six hours and then subjected to one hour ischemia and six hours of reperfusion. Serum ALT (a) IL-6 (b) and TNF-α (c) levels were determined. #P＜0.05 versus vehicle control.

**Figure S5.** Lean and ob/ob mice were subjected to one hour ischemia and six hour reperfusion. (a) Quantification of quantitative PCR of autophagy related protein mRNAs were shown. Autophagy related proteins expression (b), caspases and Lamp 2 expression (c) and was determined by western blotting. *P＜0.05 versus the lean group.

**Figure S6.** Ob/ob mice were pretreated with calpain inhibitor III (10mg/kg) or vehicle DMSO for six hours (a), or ob/ob mice were transduced with Ad-shCalpain2 or control virus (Ad-NC; 2×10^11^vp/mice) for seven days respectively (b), and then subjected to one hour ischemia and six hours of reperfusion. Liver Atg3 and Atg7 expression levels at each group were determined by western blotting. Quantification of relative protein level is shown (n=3 per group). (c) Hepatocytes isolated from ob/ob mice were subjected to four hours of anoxia and 20 min of reoxygenation in the presence or absence of calpain inhibitor III (25uM) pretreatment for one hour. Atg3, Atg4B, Atg7 and calpian 2 expression levels were determined by western blotting. Quantification of the relative protein level is shown (n=3 per group). (d) Hepatocytes isolated from ob/ob mice infected with Ad-shCalpain2 or control virus (Ad-NC) for 48 hours and then subjected to four hours of anoxia and 20 min of reoxygenation. Quantification of the relative protein level is shown (n=3 per group). ^#^P＜0.05 versus vehicle controls or Ad-NC control group, ^*^P＜0.05 versus the anoxia 0 hour group.

**Figure S7.** Ob/ob mice were transduced with Advector, AdAtg7 or AdAtg3 for seven days respectively, and then subjected to one hour of ischemia and six hours of reperfusion. Serum ALT (a), IL-6 (b) and TNF-α (c) levels were determined. #P＜0.05 versus Advector administration group.

**Figure S1**


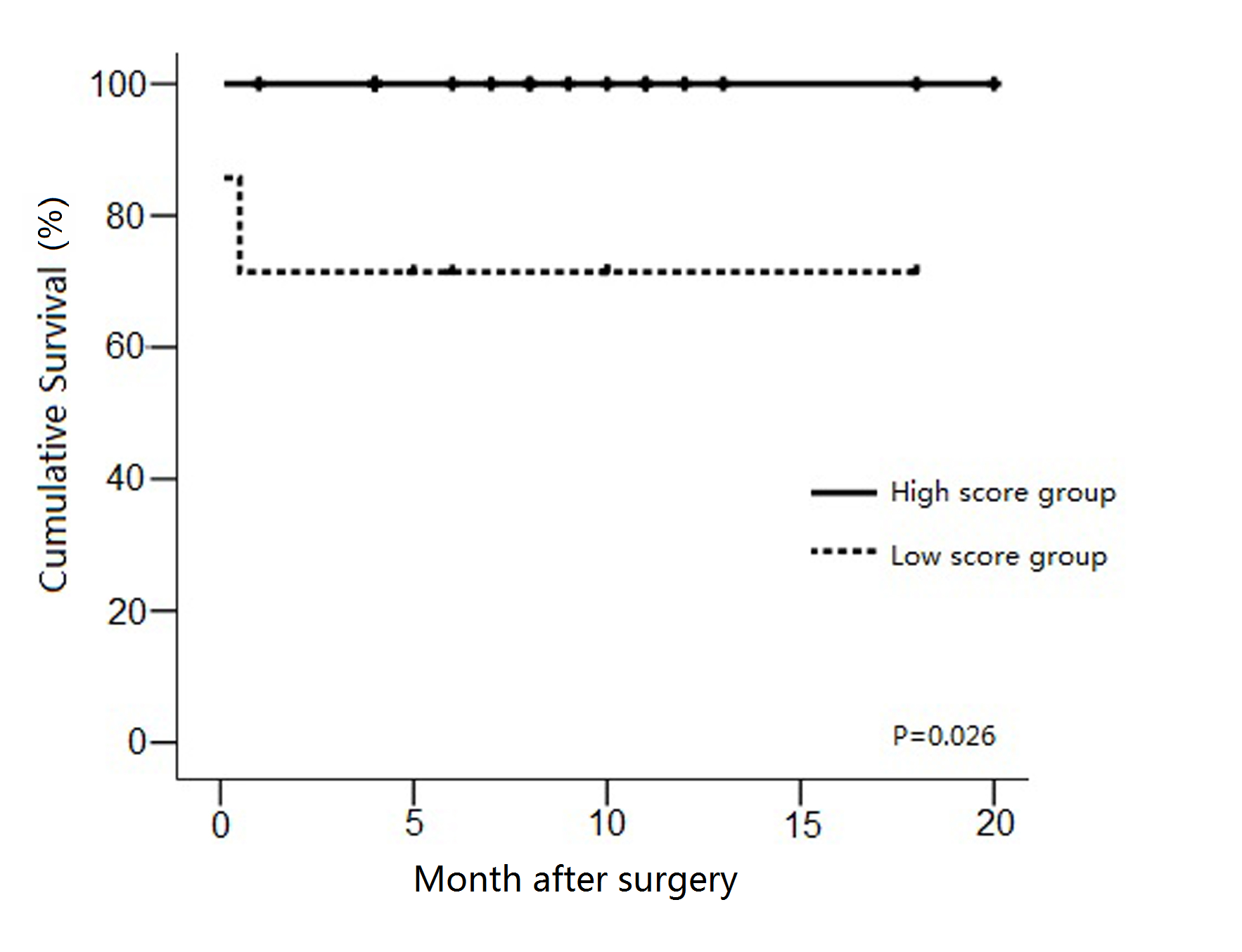


**Figure S2**


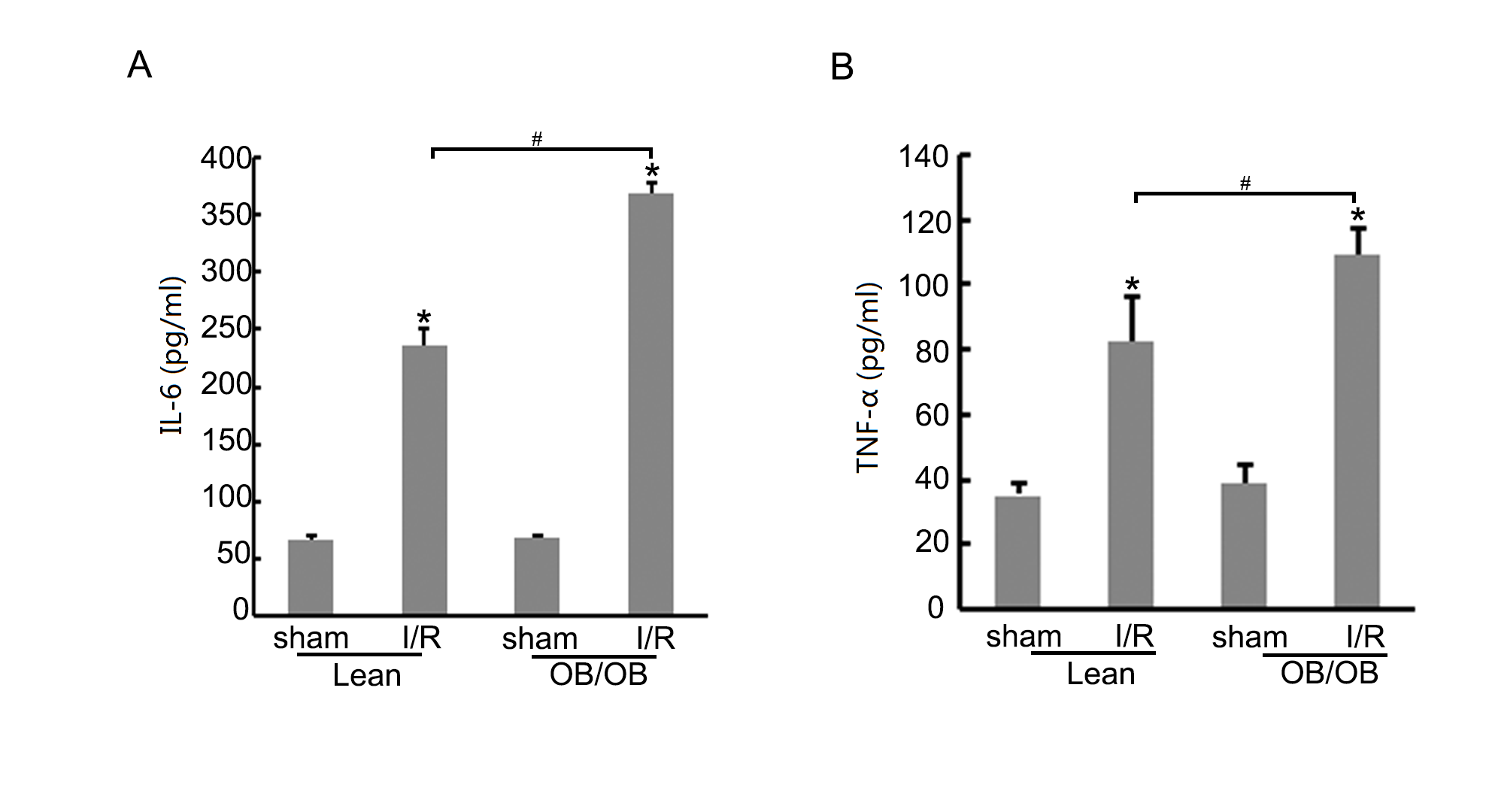


**Figure S3**


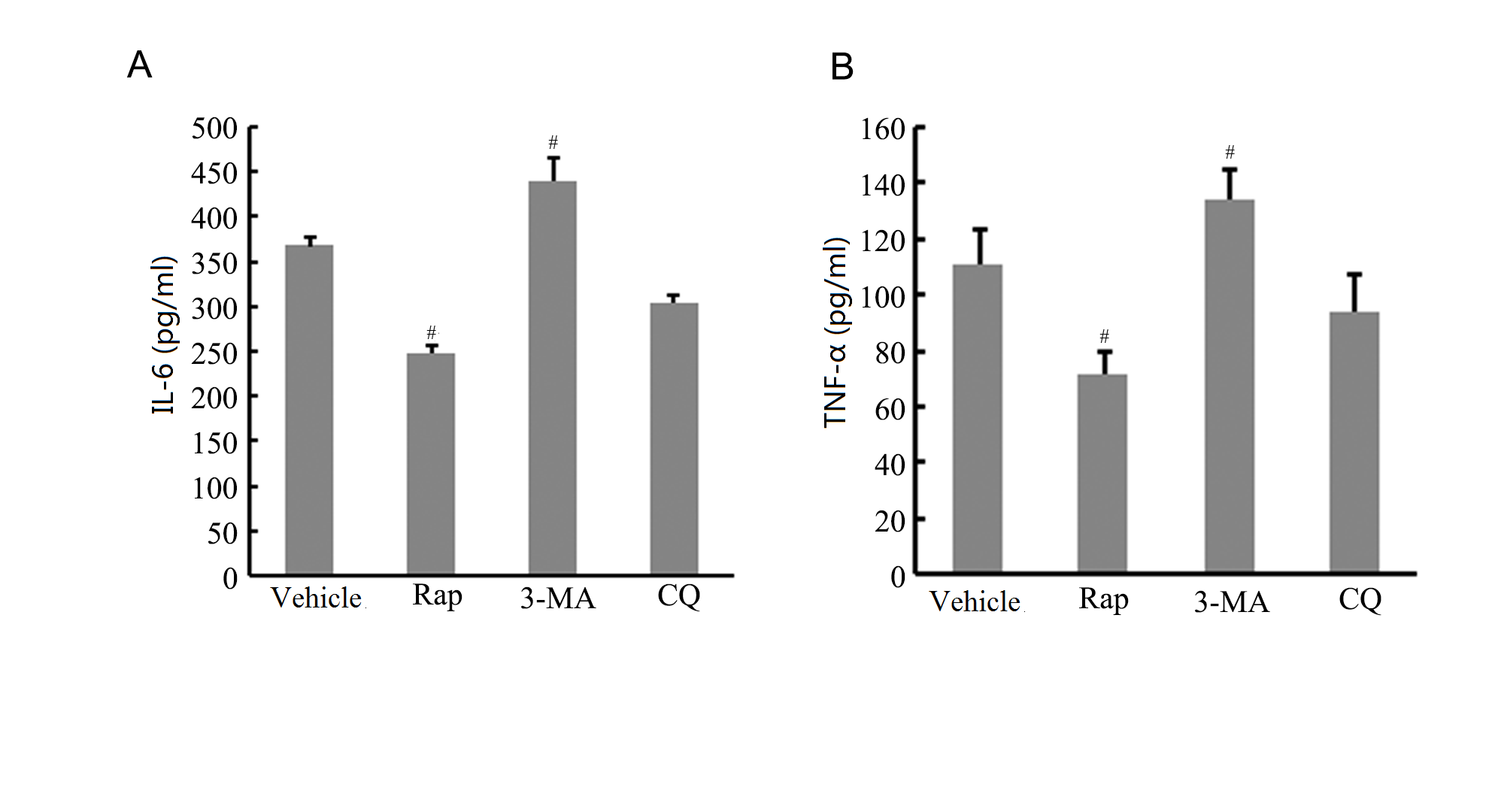


**Figure S4**


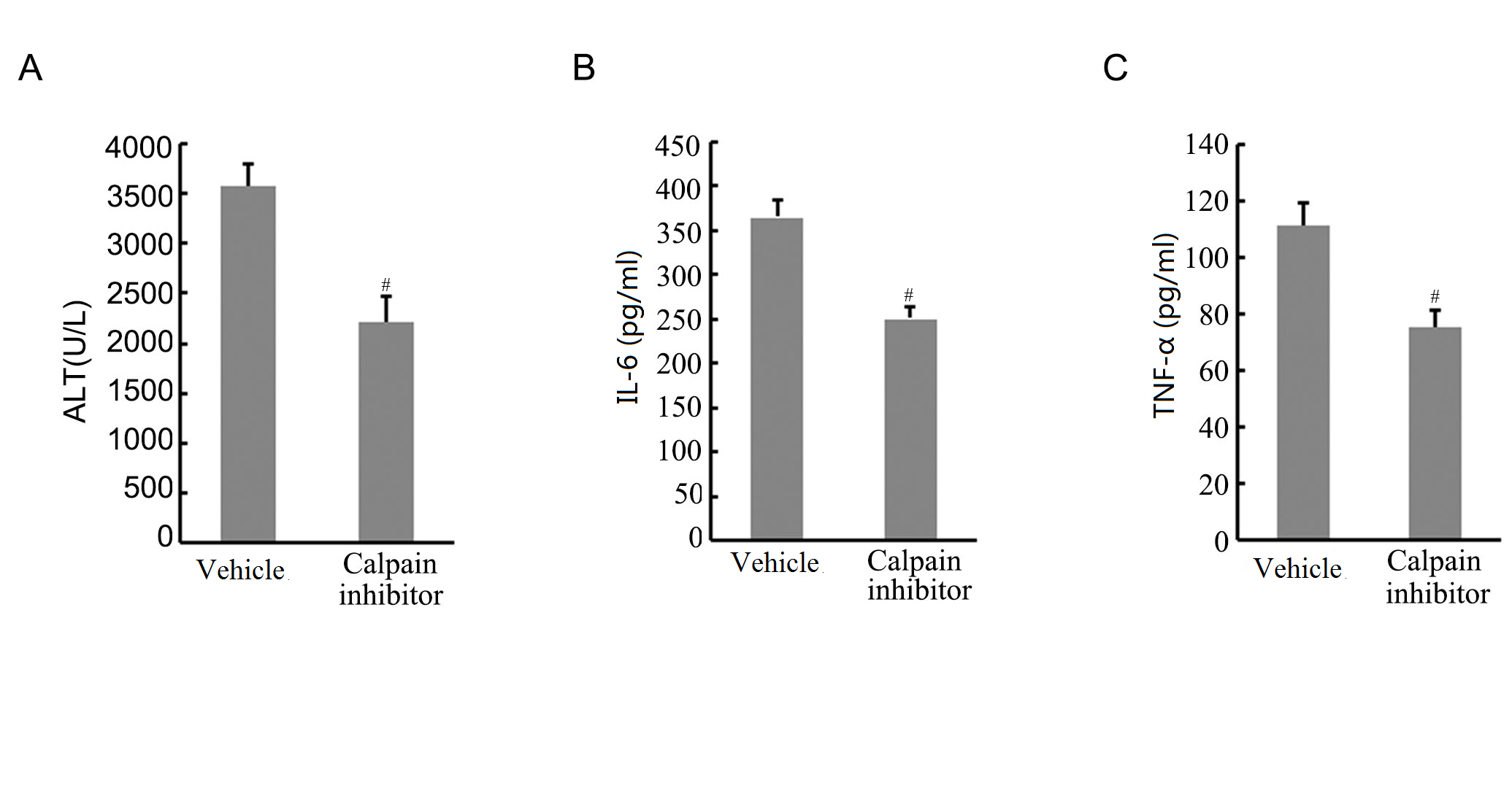


**Figure S5**


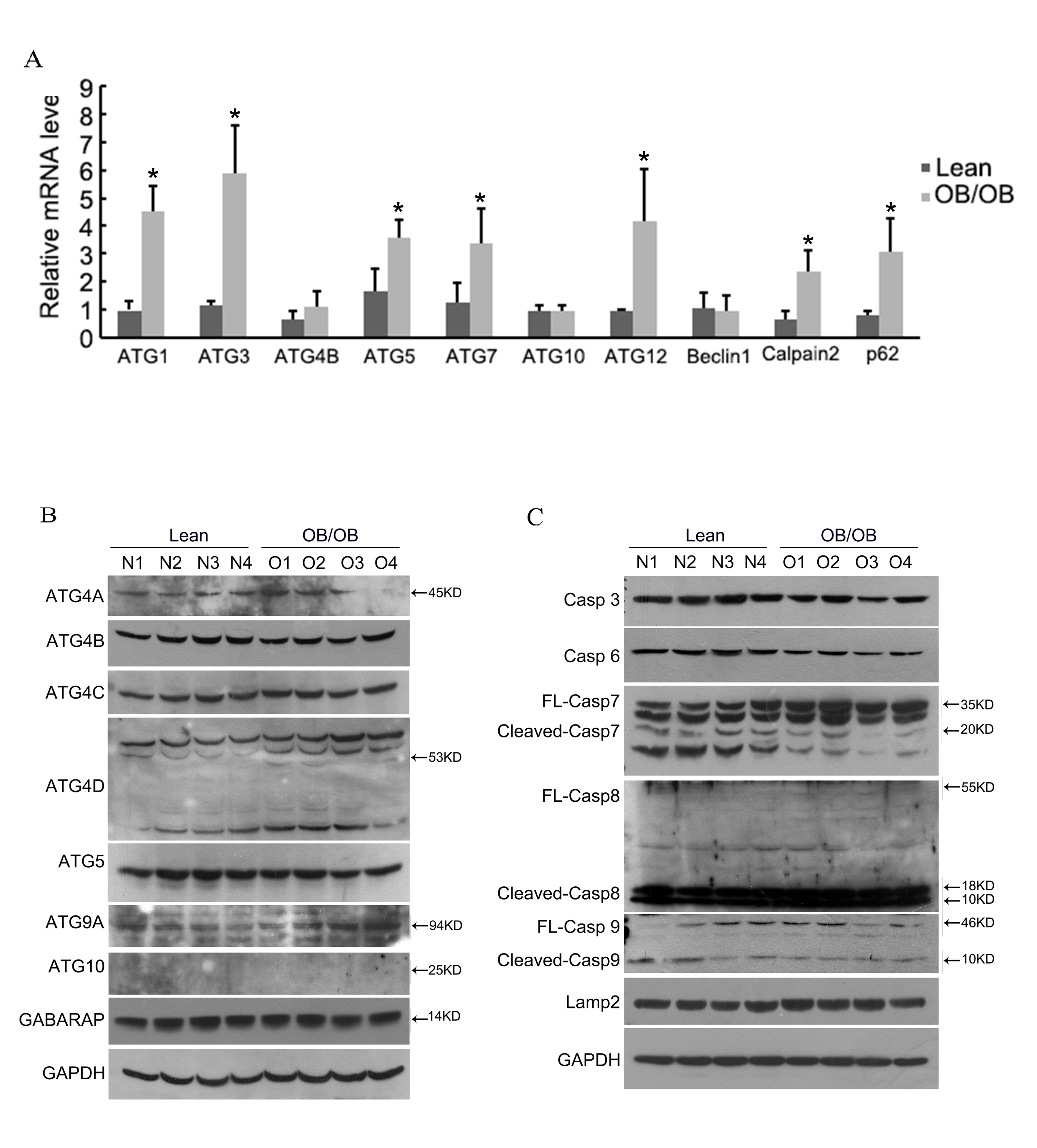


**Figure S6**


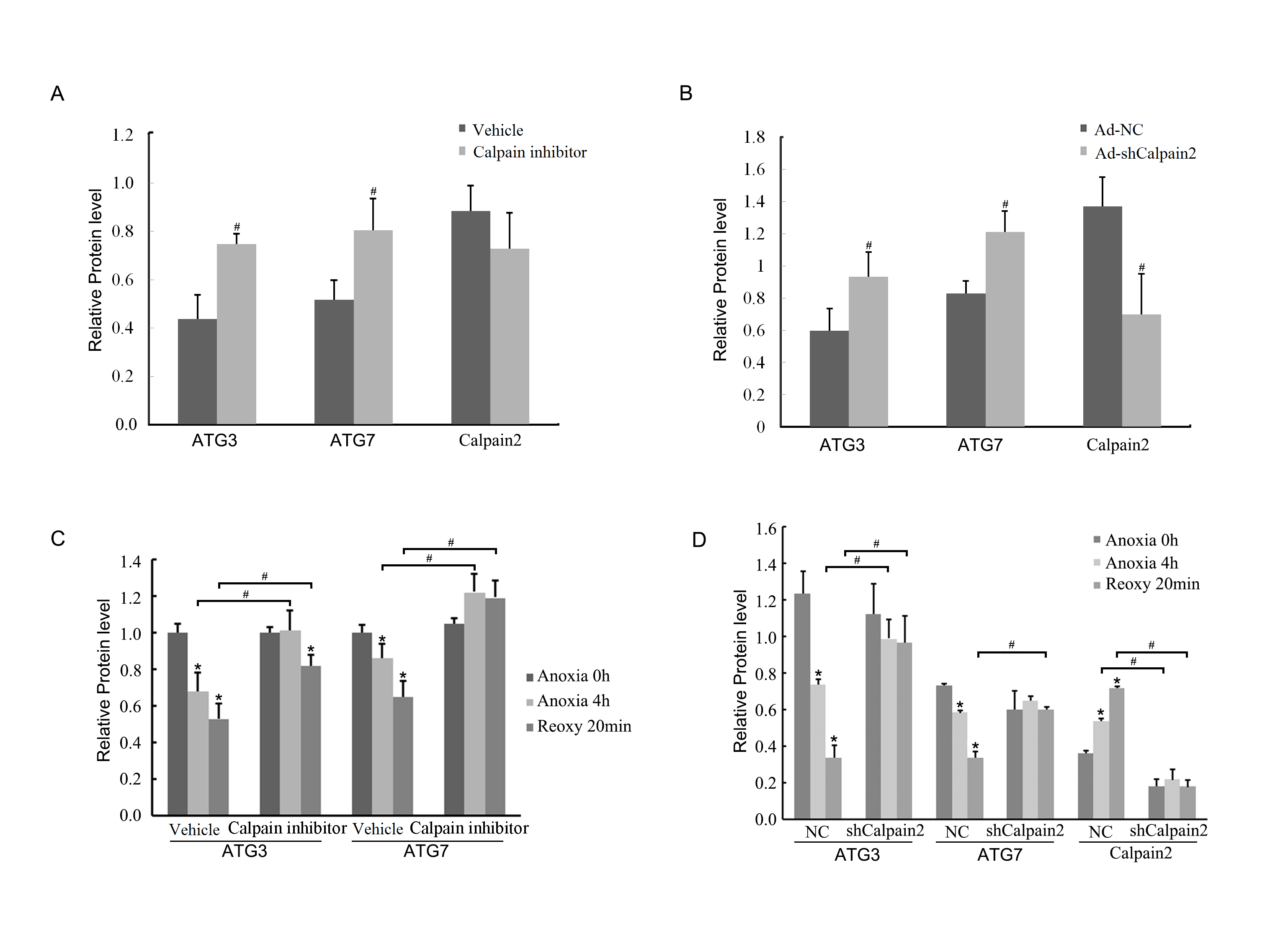


**Figure S7**


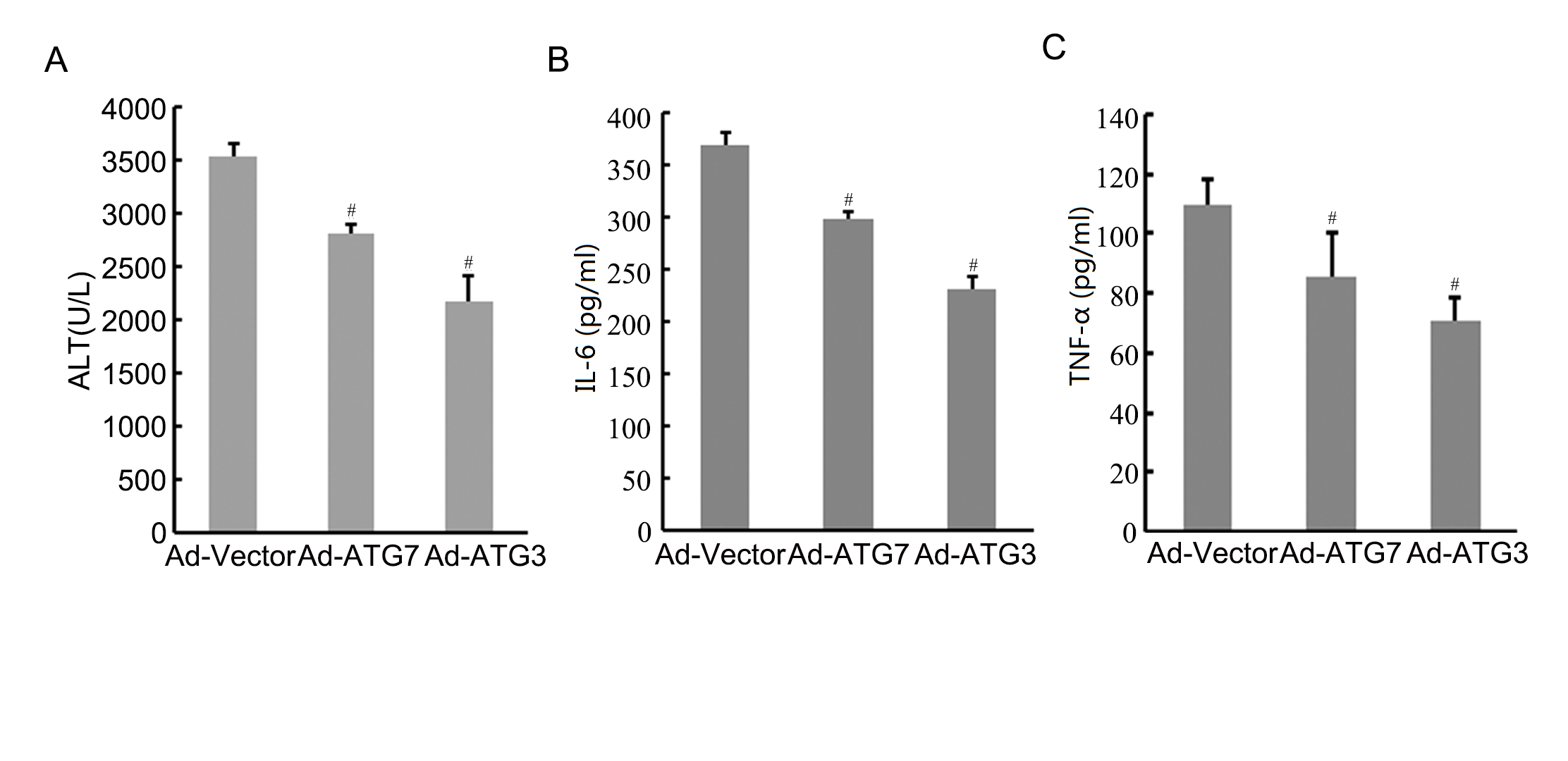

Supplement: Supplementary Information [file cddis201666x1.doc]
